# Semi-automated quantitative analysis of the middle limiting membrane in tubercular serpiginous-like choroiditis using swept-source optical coherence tomography

**DOI:** 10.1038/s41598-021-02894-9

**Published:** 2021-12-06

**Authors:** Aniruddha Agarwal, Gagan Kalra, Rupesh Agrawal, Reema Bansal, Vishali Gupta

**Affiliations:** 1 Advanced Eye Center, Post Graduate Institute of Medical Education and Research (PGIMER), Chandigarh, India; 2 Eye Institute, Cleveland Clinic Abu Dhabi (CCAD), Abu Dhabi, UAE; 3 Cole Eye Institute, Cleveland Clinic, Cleveland, OH USA; 4 National Healthcare Group Eye Institute, Tan Tock Seng Hospital, Singapore, Singapore; 5 Singapore Eye Research Institute, Singapore, Singapore; 6 Moorfields Eye Hospital, NHS Foundation Trust, London, UK; 7 Lee Kong Chian School of Medicine, Nanyang Technological University, Singapore, Singapore; 8 Duke NUS Medical School, Singapore, Singapore; 9 Yong Loo Linn School of Medicine, National University of Singapore, Singapore, Singapore; 10 School of Material Science and Engineering, Nanyang Technological University, Singapore, Singapore

**Keywords:** Eye diseases, Biomarkers

## Abstract

To analyze the longitudinal changes in the outer plexiform layer (OPL) in patients with tubercular serpiginous-like choroiditis (TB SLC) and compare it to the healthy control population. Clinical and imaging data of subjects with TB SLC (minimum 6-month follow-up) and healthy control subjects were reviewed. Optical coherence tomography (OCT) imaging obtained using swept-source device (DRI Triton, Topcon, Japan) from three visits (baseline, 3 months, and 6 months) were analyzed. Three OCT scans were chosen—one passing through the center of the fovea, one line above, and one line below. After random indexing to anonymize the images, they were pre-processed and fed into an automated pipeline to identify, crop, and measure the area of the OPL in the line scan. Longitudinal comparisons of OPL within the patient group were performed. The study included 32 eyes (16 patients; 11 males; mean age: 32.9 ± 7.8 years) with TB SLC. Twenty-eight eyes (14 subjects; 10 males: mean age: 31.1 ± 6.2 years) of healthy control subjects (age- and gender-matched) were also selected. The area of OPL was significantly different between the baseline and month 6 visit (6288 ± 1803 versus 5487 ± 1461; *p* = 0.0002) at the central scan passing through the fovea. For the scans above and below the fovea, the reduction in OPL area was significant at each visit (*p* < 0.0001). In comparison with healthy control subjects, OPL area values in patients with TB SLC were significantly lower at the month-3 (6116 ± 1441 versus 7136 ± 2539; *p* = 0.04) and the 6-month visit (5487 ± 1461 versus 7136 ± 2539; *p* < 0.001). The atrophied OPL at month 6 has been referred to as the “middle limiting membrane” (MLM). Subjects with TB SLC may develop progressive atrophy of the OPL resulting in formation of MLM, which is seen as a hyper-reflective line replacing the OPL. The analysis of longitudinal changes in the OPL may be useful in predicting anatomical and functional outcomes in these patients.

## Introduction

Tubercular serpiginous-like choroiditis (TB SLC) is common and characteristic phenotype of ocular tuberculosis, particularly in patients of Asian and Middle Eastern ethnicity from countries endemic for the disease^1–4^. TB SLC presents with unifocal or multifocal yellow-white lesions which typically have an active serpentine edge with central healing^5–7^. The lesions primarily involve the retinal pigment epithelium (RPE) and the choriocapillaris as demonstrated by histopathological studies and fundus imaging techniques such as optical coherence tomography (OCT), fundus autofluorescence (FAF), and OCT angiography (OCTA)^4, 6–8^.

Structural changes involving the choroid, RPE, and photoreceptors and alterations of inner choroidal perfusion have been studied in detail in TB SLC, less is known about how the disease involves the more superficial plexiform and nuclear retinal layers. Serial assessment of OCT B-scans from patients with TB SLC from our cohort revealed changes in the outer plexiform layer (OPL), prompting us to investigate these pathologic alterations further. Decades ago, Fine and Zimmerman performed a meticulous analysis of the outer plexiform layer (OPL) using the electron microscope^9^. The OPL is traditionally divided into three zones including the outer layer of photoreceptor axons and Müller fibers (presently termed as the Henle’s fiber layer; HFL), middle synaptic band, and inner interweaving neurons synapsing with the inner nuclear layer. Fine and Zimmerman proposed that the inner two synaptic layers be termed as “middle limiting membrane”, since this layer restricts the passage of fluid and exudates and acts as a membrane^9^.

In this study, we analyzed the OPL (“middle limiting membrane”) in serially acquired OCT studies from patients with TB SLC receiving treatment and compared these findings to those from normal healthy control subjects using objective semi-automated quantitative metrics.

## Materials and methods

This retrospective study was undertaken after approval from the Institutional Ethics Committee (IEC) of Post Graduate Institute of Medical Education and Research (PGIMER), Chandigarh, India. Since this was a retrospective study, the need for informed consent was waived off by the IEC of PGIMER. This study adheres to the declaration of Tenets of Helsinki. Subjects with newly diagnosed TB SLC were included in the analysis. The diagnosis was established based on clinical history, examination, imaging, and laboratory investigations. The criteria used for diagnosing TB SLC were^1–3^:

A. Presence of serpiginous-like choroiditis lesions in the posterior pole.
B. Exclusion of other uveitis entities
C. One or more positive corroborative investigations including chest radiography or contrast-enhanced computerized tomography of the chest (CT-scan) consistent with TB infection, clinical evidence of extraocular TB, microbiological confirmation from sputum or extraocular sites *along with* immunological evidence of TB (tuberculin skin test: positive induration of 10 × 10 mm after 48–72 h or positive interferon gamma release assay (IGRA) test: positive QuantiFERON of > 0.35 IU/ml).

Patients with less than 6 months of follow-up duration were excluded. Eyes with media opacities or poor fixation precluding adequate image quality were excluded. Patients who developed paradoxical worsening after initiation of anti-tubercular therapy (ATT) and corticosteroids were also excluded from the analysis. None of the patients received intravitreal therapies. Three visits with OCT imaging data and complete clinical details were mandatory for patient inclusion in the study. The 3 time points analyzed were the earliest encounter/baseline (M1), encounter at 3 months after initiation of therapy (M2) and the follow-up visit at 6 months (M3). All patients underwent complete ocular examinations including Snellen best-corrected visual acuity (BCVA), slit-lamp biomicroscopy and indirect ophthalmoscopy as well as ocular imaging including color fundus photography (Visupac FF450, Carl Zeiss Meditec, Jena, Germany) and SS-OCT (DRI Triton, Topcon, Japan).

For the analysis, OCT data from eyes of healthy control subjects was used for the analysis of quantitative parameters derived from OCT imaging. Control subjects had no evidence of ocular or systemic disease and underwent detailed ophthalmic examination and refraction. Control eyes were included only if Snellen BCVA was ≥ 20/20 with a refractive error of no more than ± 3.0.

**Figure 1:**
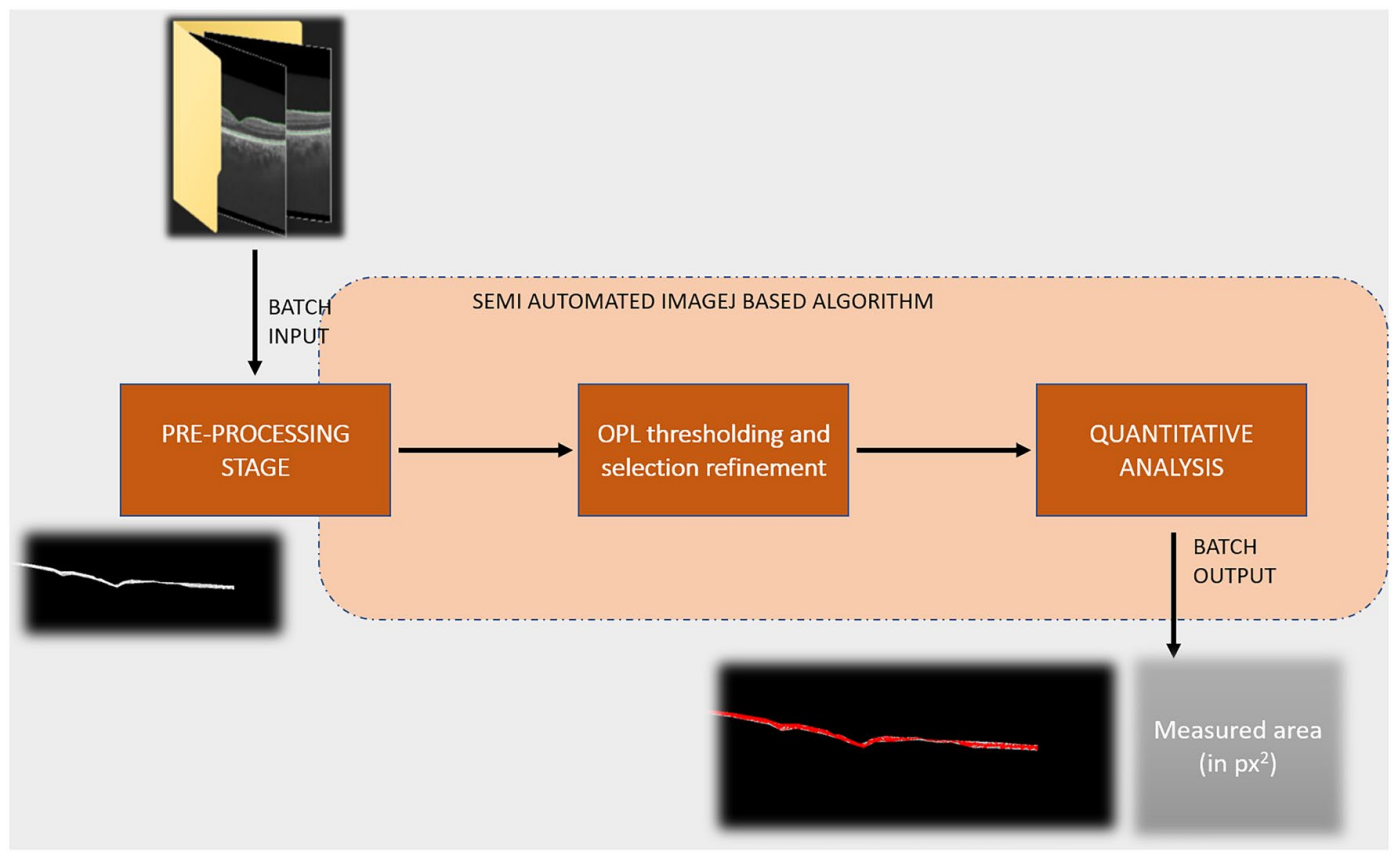
The figure shows the algorithm used in the semi-automated quantitative analysis of the outer plexiform layer (OPL) area in the included subjects in our study. The pre-processing stage consisted of randomization, anonymization and cropped using third party software (ImageJ). The subsequent automated stages included conversion to 8-bit and thresholding, and measurement of the area in square pixels.

All the SS-OCT images of the three visits for each patient were randomly labelled and indexed to avoid any bias. Normal control subjects only underwent OCT at one baseline visit. The grader was masked to the subject identity and time point. All the patients underwent 5-line raster scanning on the OCT, with a fixed B-scan length of 6 mm for all eyes included in the study. Three SS-OCT line scans were chosen for analysis at each visit: one line scan passing through the center of the fovea, one line above and one below the fovea. The lines above and below the fovea were 300 microns apart. All image analysis were performed using ImageJ (version1.53c, National Institute of Health, Bethesda, USA) and automation was performed using the macro-JAVA capability within ImageJ (Fig. 1).

In a pre-processing stage (manual phase), all the SS-OCT images were randomized, anonymized, and cropped using freehand selection tool in ImageJ using the inner nuclear layer and outer nuclear layer as cropping boundaries to grossly isolate the OPL. The resulting image from this stage would be subsequently referred to as pre-processed image in the manuscript. The pre-processed images from the left and the right eyes were fed into the subsequent pipeline separately.

**Figure 2:**
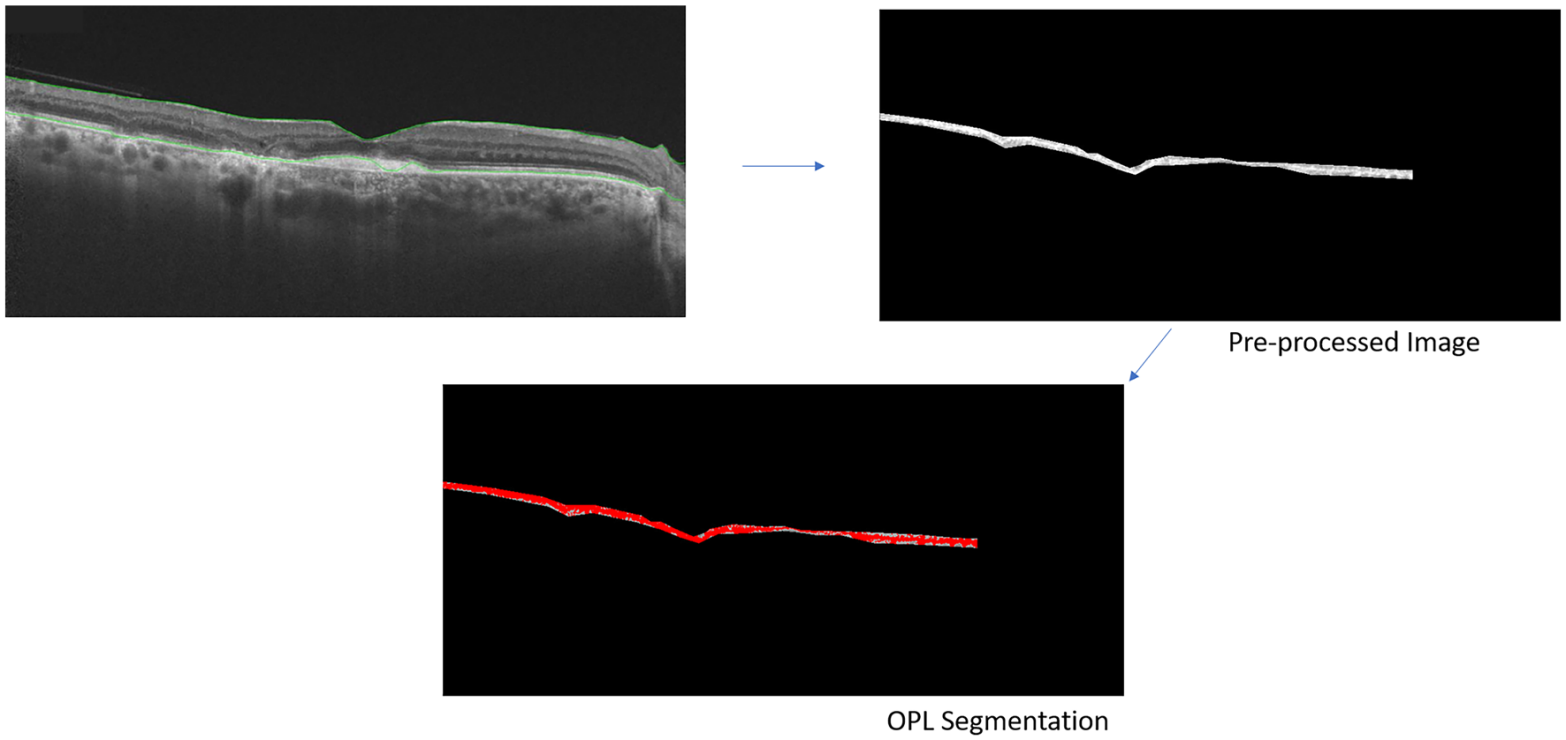
Illustrates the segmentation and cropping of the outer plexiform layer from the optical coherence tomography line scan, and subsequent thresholding for calculation of the area using an automated algorithm on ImageJ.

In the analysis phase (automated phase) (Fig. 2), the pre-processed image was converted to 8-bit, a rectangular selection of width 230 pixels and 260 pixels in height, on the right extreme in the images from the right eye and the left extreme in the images from the left eye, was excluded from the analysis and an automated threshold was applied to the image to precisely isolate and select the OPL. This rectangular area was cropped so that the peripapillary retina could be excluded from the analysis, where identification of the layers may be challenging. Subsequently the area of the OPL (in pixels) was measured and used as an output metric.

Data analysis was performed using GraphPad Prism (GraphPad Software Inc., La Jolla, CA) version 6.0 (https://www.graphpad.com/scientific-software/prism/). Demographic data was expressed in mean and standard deviation. The measured area of the OPL was compared between the three visits using one-way repeated measures analysis of variance (ANOVA) and post-hoc Tukey’s honestly significant difference (HSD) test. The comparisons of OPL area between patients at various visits and the normal subjects was performed using Mann–Whitney U test. Generalized linear model was used to address the dependency of two eyes per patient. The best-corrected visual acuity was correlated with the change in OPL area measurements using the Pearson’s correlation test. The statistical significance was denoted by *p* < 0.05 and 95% confidence interval.

## Results

In this study, 16 patients (32 eyes; 11 males and 5 females) with TB SLC were included. All the subjects were Asian Indian in ethnicity. The mean age of all the subjects was 32.94 ± 7.84 years. Based on the pre-decided exclusion criteria, subjects with less than 6 months of follow-up were excluded from the analysis. Fourteen age- and gender-matched normal subjects (28 eyes; 10 males) were included in the analysis. The mean age of the normal control subjects was 31.07 ± 6.23 years. The demographic characteristics and the ongoing therapies have been described in Table 1.

**Table 1:** Demographic and clinical characteristics of patients with tubercular serpiginous-like choroiditis and normal healthy control subjects included in the study.

Variable	Patients with TB SLC	Normal Healthy Controls	*p* value
Number of patients (n)	16	14	–
Age (years ± SD)	32.94 ± 7.84	31.07 ± 6.23	0.60
**Gender**			
Male (n)	11	10	0.87
Female (n)	5	4	
Duration of symptoms (weeks)	3.81 ± 2.29	–	–
Baseline BCVA (LogMAR)	0.36 ± 0.21	0.07 ± 0.08	0.0003
Investigations		–	–
Positive Mantoux* (n)	13		
Positive IGRA** (n)	8		
Positive CT Chest† (n)	6		
Positive biopsy‡ (n)	2		
Treatment		–	–
ATT and systemic corticosteroids (n)	16		

*Indicates induration ≥ 10 mm × 10 mm by tuberculin skin test after 48–72 h.

**Indicates a positive interferon gamma assay (IGRA) using QuantiFERON TB Gold® test.

^†^Indicates evidence of healed or active tuberculosis on computerized chest tomography.

^‡^Indicates biopsy from lung/lymph nodes, or minor salivary glands.

The area of the OPL (in pixels) among patients in the line-scan at the macula, line above the macula and line below the macula (at the three visits: M1, M2 and M3) have been summarized in Table 2. The OPL layer area at the macula analyzed using one-way repeated measures ANOVA with post-hoc Tukey’s HSD test revealed that the area was significantly lower at visit M3 compared to visit M1 (*p* = 0.0002), whereas no significant differences existed between visits M1-M2 or M2-M3. Similar analysis on the line-scan above the macula revealed significant reduction in OPL area at each visit (all *p* < 0.0001). Line-scan below the macula also showed significant reduction in OPL area at each consecutive visit (*p* < 0.0001). The OPL values and their comparisons have been depicted in Table 2.

**Table 2:** The mean area (± standard deviation) of the outer plexiform layer (OPL) among patients with tubercular serpiginous-like choroiditis (TB SLC) measured using semi-automated quantitative techniques on optical coherence tomography line scans.

	Baseline visit (M1)	Month 3 (M2)	Month 6 (M3)	*p* value
Macula	6288 ± 1803	6116 ± 1441	5487 ± 1461*	0.0002
Above	7614 ± 1509	6629 ± 1475*	5559 ± 1516* †	< 0.0001
Below	7287 ± 1784	6147 ± 1735*	5176 ± 1655* †	< 0.0001

The data is represented in mean pixel square (area) of the outer plexiform layer.

The analysis is performed using one-way repeated measures analysis of variance (ANOVA).

*p < 0.05 vs baseline visit (using post-hoc Tukey’s test).

^†^*p* < 0.05 vs month 3 visit (using post-hoc Tukey’s test).

**Figure 3:**
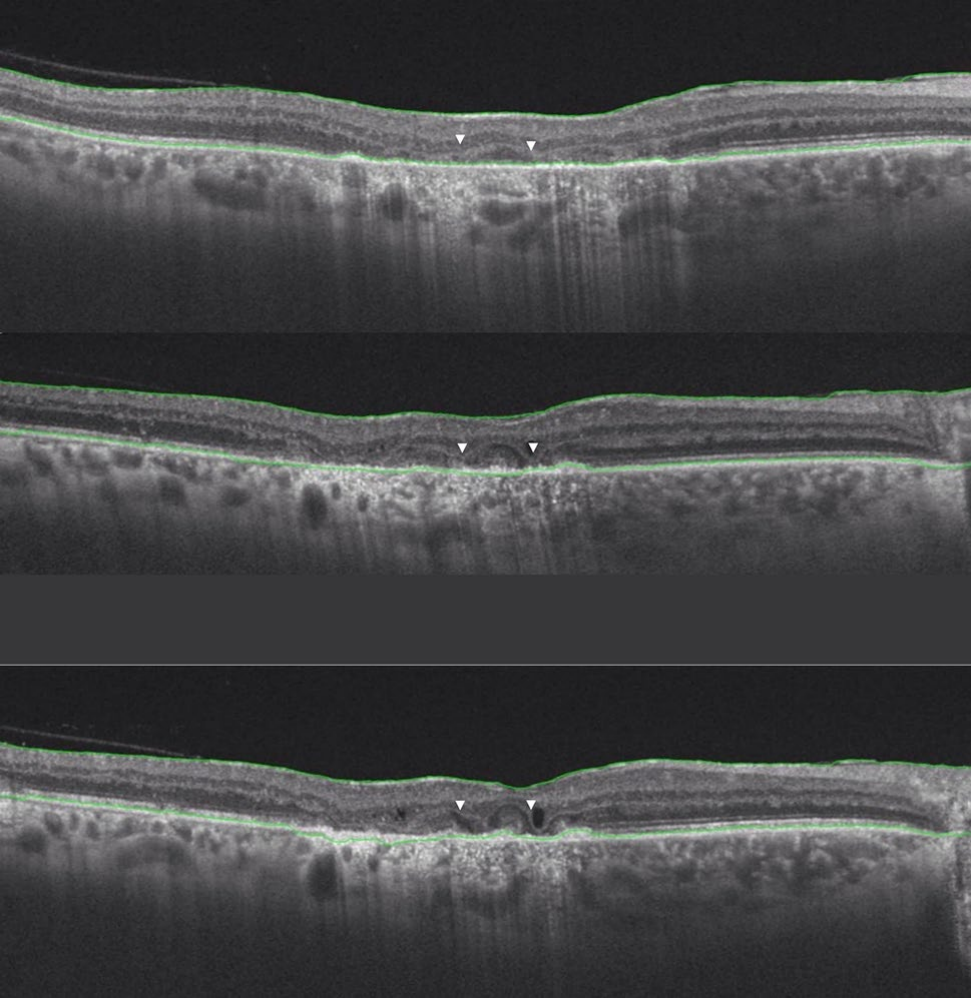
A representative figure of a patient with tubercular serpiginous-like choroiditis showing foveal optical coherence tomography (OCT) B-scans at three visits. The foveal OCT B-scan at first visit (top panel) shows a near-normal hyper-reflective outer plexiform layer (OPL). The foveal OCT B-scan at second visit (3 months) shows thinning of the OPL, which has been thrown into undulations (middle panel). The OCT B-scan at third visit (6 months) (bottom panel) shows significant thinning and atrophy, resulting in formation of a middle limiting membrane (white arrowheads).

Figure 3 shows progressive reduction in the OPL area in a patient with TB SLC leading to formation of “middle limiting membrane”.

In comparing normal healthy control subjects with patients, the OPL area was not significantly different from patients with TB SLC at baseline (M1). However, the OPL area measurements among patients with TB SLC were significantly lower compared to normal control subjects at M2 and M3 visit in the OCT through the fovea and below the fovea (*p* < 0.04). The OPL area was significantly lower in patients compared to healthy controls at only the M3 visit in the OCT line-scan above the fovea (*p* < 0.001). The details of comparisons between patients and healthy control subjects are depicted in Table 3.

**Table 3:** Comparison between the area of outer plexiform layer (OPL) (in mean pixel square) between patients with tubercular serpiginous-like choroiditis and normal healthy control subjects.

	Patients (n = 32 eyes)	Normal control (n = 28 eyes)	*p* value
**Fovea**			
M1	6827.75 ± 1803.51	7135.93 ± 2538.57	0.75
M2	6115.53 ± 1440.71	–	**0.04**
M3	5486.5 ± 1461.09	–	** < 0.001**
**Above fovea**			
M1	7613.66 ± 1509.18	7513.04 ± 2822.76	0.35
M2	6628.97 ± 1474.79	–	0.11
M3	5558.97 ± 1515.95	–	** < 0.001**
**Below fovea**			
M1	7286.66 ± 1784.21	7517.79 ± 2519.80	0.73
M2	6146.91 ± 1734.83	–	**0.01**
M3	5176.28 ± 1654.71	–	** < 0.001**

M1: Baseline visit.

M2: Intermediate visit at month 3.

M3: Final visit at month 6.

The *p* value was calculated by using Mann–Whitney U test.

Significant values are in bold.

The BCVA was 0.9 ± 0.26 LogMAR units at M1 and 0.52 ± 0.4 at M3 visit (*p* < 0.001). The BCVA at M1 showed a fair correlation with the reduction in OPL area (between visits M1 and M3) (r = 0.33; *p* = 0.04).

## Discussion

The observations from histopathological studies by Fine and Zimmerman several decades ago have shed light on the ultrastructural details of the OPL^9^. The “bands” of the OPL consists of retinal cells which are tightly interwoven with multiple synapses and desmosomal connections. The three zones of OPL bands include: outer broad layer of photoreceptor axons and outer halves of Muller cells; middle narrow synaptic band; and third inner band of neurites between the synaptic middle band and the inner nuclear layer. The inner two narrow bands form a “limiting membrane” because of their inherent ultrastructural nature consisting of interwoven synapsis and cell junctions (desmosomes)^9^.

The concept of “prominent middle limiting membrane (p-MLM)” due to ischemic retinal insult was introduced in 2013 by Chu et al.^10^, who speculated that cytoplasmic swelling of the bipolar cells in the inner OPL resulted in OCT appearance of a hyper-reflective line in patients with acute retinal ischemia due to central retinal arteriolar occlusion. The p-MLM sign has also been observed in other conditions such as central retinal vein occlusion along with paracentral acute middle maculopathy (PAMM)^11–13^.

The OPL layer is highly oxygen-dependent and is located at the outer limits of the retinal perfusion^14, 15^. The outer region of the OPL derives its vascular supply from the choroid. Therefore, this layer appears to be prone to both retinal and choroidal vascular compromise resulting from a number of pathological conditions. Retinal vascular occlusions cause rapid-onset diminution of blood supply resulting in acute injury to the cellular structures in OPL^10, 13^. However, in TB SLC, a compromised choriocapillaris perfusion^4, 5, 16^ results in ischemic damage to the OPL. This is seen as a gradual decrease in the OPL thickness on the OCT (as seen in our study). Eventually, complete atrophy of cellular structures in the OPL results in a linear hyper-reflective line instead of the OPL. This residual layer probably consists of remnant extracellular structures such as desmosomal proteins (Fig. 3). The hyper-reflective OPL line represents the “middle limiting membrane” in TB SLC, which is structurally and pathologically different from the p-MLM in acute retinal ischemia. Similar morphological changes in the OPL have been observed on histopathological analysis using in vivo rat models following prolonged ischemia. In these specimens, authors have observed atrophy and thinning of the OPL with the destruction of neuronal cells^17^.

The OPL has also been shown to decrease in thickness with age and disease^18^. However, the reduction in OPL thickness is an important feature of early age-related macular degeneration (AMD)^18–22^. Thinning of the OPL may precede development of drusen^23^. The changes in OPL may occur due to early and progressive neurodegeneration in addition to neuronal migration and peripheral (extramacular proliferation) in AMD^18, 19^. Similar mechanisms of OPL remodeling may occur in TB SLC, resulting in thinning of the OPL layer and formation of the MLM. However, unlike in AMD, histopathological data on neurodegeneration and neuronal remodeling in TB SLC is not available in the literature.

Detection of the middle limiting membrane in TB SLC may be useful in improving our understanding of the pathophysiological mechanisms involved in ocular inflammation. Middle limiting membrane in TB SLC signifies permanent retinal structural damage from the disease. Inflammation in TB SLC is primarily located in the choriocapillaris and the RPE. However, the consequences of choriocapillaris ischemia in TB SLC can have effects reaching up to the OPL and beyond, besides involving the RPE, photoreceptor and choriocapillaris. These structural changes may have adverse functional impact resulting in reduction of synaptic conduction in areas affected by choriocapillaris ischemia and may highlight the need for prompt and aggressive therapy to minimize damage to middle retina in addition to outer retina, RPE-choriocapillaris complex. We observed that presence of worse baseline BCVA was associated with a greater reduction in the OPL area measurements in comparing the baseline and final visit values. Thus, measuring the OPL area may have functional significance as it shows a fair correlation with the baseline BCVA. However, it is difficult to comment whether identifying the middle limiting membrane would enhance one’s ability to diagnose, prognosticate or treat these patients. However, it is a novel finding that needs to be studied further.

In our study, we used an objective method to calculate the OPL area in three OCT line-scans encompassing the foveal center. This was performed to avoid subjective identification of “middle limiting membrane” unlike previous studies in retinal vascular occlusions^10–13^. In addition, the area was calculated in pixels to avoid errors due to conversion to metric units. Moreover, we calculated the area of the OPL (rather than layer thickness) across the entire OCT line scan so that it was more representative of global OPL changes. Measuring OPL thickness at fixed distances from the foveal center may result in missed data points, leading to erroneous calculations.

A semi-automated method of delimiting the OPL from the original OCT image resulted in rapid assessment of multiple images while minimizing the risk of measurement bias. Since we included normal control subjects and obtained their OPL area measurements, comparisons could be made at various visits, and for different line scans. Our results showed that there was no significant difference at baseline in the OPL area at all three scan locations between patients with TB SLC and normal control subjects. However, during the course of the disease, the OPL area among patients with TB SLC significantly reduced compared to baseline especially at month 6 (Table 2) and was significantly lower compared to healthy control subjects in the follow-up (Table 3).

The results of our study reveal the importance of identifying retinal structural changes in patients with TB SLC even though it is primarily an RPE-choriocapillaris inflammation. Retinal structural changes may also result in future complications such as development of type 2 choroidal neovascularization due to the ischemic changes.

Our study has several limitations. Since it is a retrospective study, there could be sources of bias such as selection bias. However, we included all consecutive eyes with TB SLC without complications such as paradoxical worsening or choroidal neovascularization. Subjects receiving intensive therapies such as intravenous methylprednisolone or intravitreal agents, and immunosuppressive therapies were excluded from the analysis. None of the patients had previously failed treatments or concomitant retinal diseases that could affect the OPL. The measurements were performed using semi-automated techniques with images de-identified in order to avoid measurement bias (Figs. 1 and 2). We observed large standard deviations in the measurements of the OPL area (Table 2). These were due to the variations in the thicknesses of the OPL between eyes. Since we used an automated pipeline to measure the OPL area, repeated measurements would not alter the magnitude of the standard deviations.

The Henle fiber layer (HFL) consists of photoreceptor axons and Müller cell processes which have unique directional and optical properties^24^. The HFL appears iso-reflective to the outer nuclear layer (ONL) on OCT, leading to its erroneous inclusion in ONL thickness layer measurements^25–28^. Directional OCT is a technique to overcome the challenges posed by the HFL by altering the incident beam entry position^29^. We did not use directional OCT in our study, and this represents a limitation in our study. However, HFL greatly impacts the ONL thickness measurements, but its impact on OPL layer measurements is not exactly known. While directionality and optical properties of HFL may also affect OPL measurements, further studies in this regard may help understand the magnitude of error that can be prevented by using directional OCT. Since our technique of measurement of OPL has not been widely used, larger datasets may help in validating the results of our study. Prospective studies can be undertaken to colocalize the changes in the OPL with alterations in choriocapillaris perfusion using topography-guided assessments with optical coherence tomography angiography.

In summary, subjects with TB SLC can develop pathological atrophy of the OPL during treatment resulting in formation of a “middle limiting membrane”. This finding is seen on the OCT as a hyper-reflective line instead of the OPL. Such changes involving the retinal layers in TB SLC can lead to reduced functional acuity in the long-term. Further studies are needed to evaluate the significance of such pathological alterations in ocular inflammatory conditions.
